# Reconstruction of human brain spontaneous activity based on frequency-pattern analysis of magnetoencephalography data

**DOI:** 10.3389/fnins.2015.00373

**Published:** 2015-10-16

**Authors:** Rodolfo R. Llinás, Mikhail N. Ustinin, Stanislav D. Rykunov, Anna I. Boyko, Vyacheslav V. Sychev, Kerry D. Walton, Guilherme M. Rabello, John Garcia

**Affiliations:** ^1^Department of Neuroscience and Physiology, New York University School of MedicineNew York, NY, USA; ^2^Institute of Mathematical Problems of Biology, Russian Academy of SciencesPushchino, Russia

**Keywords:** magnetic encephalography, frequency-pattern analysis, functional tomography, phantom data, alpha rhythm, inverse problem solution

## Abstract

A new method for the analysis and localization of brain activity has been developed, based on multichannel magnetic field recordings, over minutes, superimposed on the MRI of the individual. Here, a high resolution Fourier Transform is obtained over the entire recording period, leading to a detailed multi-frequency spectrum. Further analysis implements a total decomposition of the frequency components into functionally invariant entities, each having an invariant field pattern localizable in recording space. The method, addressed as functional tomography, makes it possible to find the distribution of magnetic field sources in space. Here, the method is applied to the analysis of simulated data, to oscillating signals activating a physical current dipoles phantom, and to recordings of spontaneous brain activity in 10 healthy adults. In the analysis of simulated data, 61 dipoles are localized with 0.7 mm precision. Concerning the physical phantom the method is able to localize three simultaneously activated current dipoles with 1 mm precision. Spatial resolution 3 mm was attained when localizing spontaneous alpha rhythm activity in 10 healthy adults, where the alpha peak was specified for each subject individually. Co-registration of the functional tomograms with each subject's head MRI localized alpha range activity to the occipital and/or posterior parietal brain region. This is the first application of this new functional tomography to human brain activity. The method successfully provides an overall view of brain electrical activity, a detailed spectral description and, combined with MRI, the localization of sources in anatomical brain space.

## Introduction

Magnetoencephalography (MEG) has become one of the foremost biological technologies addressing detailed analysis of human brain function and recently an open archive has been established (Niso et al., 2015). Thus, recorded magnetic fields with a high sampling rate, and hundreds of recording channels, can provide a functional image of unprecedented precision, comprising cortical as well as deep brain structures. Due to its methodological character, this approach can analyze large data sets affording the comprehensive analysis of functional detail. Concerning the human brain, two main parameters have challenged global analysis of function. One is the simultaneous nature of brain neuronal activity where, at any given instant, millions of neuronal functional events co-exist. The other is the great variety in neuronal morphologies that, upon activation, lead to the generation of different electromagnetic field profiles (Llinás, 1988). Historically, the most common approach to such a conundrum has been to address the brain activity that follows a given stimuli (evoked or induced potentials), or to address the characteristics of spontaneous (resting or ongoing) electromagnetic activity related to large events such as various sleep and waking states (Llinás and Pare, 1991).

Historically, the former approaches, i.e., the analysis of sensory evoked potentials, or those obtained from abnormal brain function relating to synchronous activation of vast number of neurons (e.g., epilepsy, Ossenblok et al., 2007) has been the most commonly addressed. However, the final results, even under such favorable conditions as the analysis of repeated simple stimuli that may be averaged, have not yielded the imaging required to address dynamic brain functional activity and this remains a field of active research (David et al., 2006a,b; Klimesch et al., 2007; Sauseng et al., 2007; Ros et al., 2015). Under those conditions, the content of moment-to-moment brain function is lost and only those aspects that relate to the common features of the given repeated stimuli are addressable.

In an attempt to move away from the evoked activity approach and toward the analysis of ongoing brain activity a new method has been developed to represent global brain activity as a set of elementary coherent oscillations (Llinás and Ustinin, 2014a,b). The core of the proposed technology lies in the performing of the precise detailed Fourier transform of the long multichannel time series and in the analysis of the frequency components obtained. Theorems were proved, stating that if phases are equal in all channels of some elementary oscillation (characterized by distinct frequency), then the normalized pattern of this oscillation is constant through the period. Mathematically it means separation of variables (time course and spatial structure are simply multiplied). It means, that such elementary coherent oscillation ideally cleans the spatial structure of the field at this particular frequency. This approach was applied to 19 experimental MEG data sets of human spontaneous activity, and it was found, that many elementary oscillations reveal high coherence and hence are representing static structures, generating corresponding frequencies. The next step was to further divide those oscillations, which are not coherent, but still look rather simple, because of the detailed frequency representation. As a result, the multichannel signal is decomposed into the set of elementary coherent oscillations. Note, that this decomposition is obtained by the direct nonparametric transformation of the initial data, it is precise and totally reversible. The solution of the inverse problem for each elementary oscillation provides the spatial structure of the source, oscillating as a whole with time course, extracted earlier. When inverse problems will be solved for all oscillations, the system under study will be represented as a sum of stable sources (functional entities), each of them oscillating as a whole. Many methods of inverse problem solution were developed (e.g., Hämäläinen and Ilmoniemi, 1994; Sekihara and Nagarajan, 2008; Kozunov and Ossadtchi, 2015). Some of these methods, especially those devised for simple source models, can be effectively used to reconstruct functional entities, extracted by the proposed technology. The fact, that proposed technology splits MEG into elementary oscillations with relatively simple patterns, can revive few-channel measurements, including those combined with MRI (Zotev et al., 2008; Cottereau et al., 2015; Fukushima et al., 2015).

Here we assume, that the considerable part of the MEG signal in the alpha rhythm frequency band can be represented as a sum of equivalent current dipoles, while each coherent oscillation is described by one dipole. In order to check this assumption, the following experiments were performed. Computer simulation (61 dipoles plus noise) and physical modeling (3 dipoles) were used as a benchmark, estimating initial data with good precision. Then the method was blindly applied to study the alpha rhythm in 10 human subjects, localizing ~2000 dipoles for each person. The alpha rhythm is often used as a benchmark for different methods (Pascual-Marqui et al., 2014), because of the relatively well-known nature of this phenomenon. The blind application of the method means here, that no anatomical information about the brain is used to solve the inverse problem. When initial MEG is split into the set of elementary coherent oscillations, inverse problem is solved for every oscillation pattern in one current dipole model and the energy of oscillation is attributed to the spatial position of the dipole. Repeating this procedure for all oscillations, one can obtain the Functional Tomogram (FT)—spatial distribution of the sources, generating the initial MEG. The allowable localization space is a 25 × 25 × 25 cm^3^ cube, and it is the geometrical size of the FT.

In order to better evaluate the method, functional tomogram is compared with individual brain anatomy only after the end of computations. It can be schematically shown as:

MEG registration → Calculation of the FT → Representation of the FT with MRI ← MRI registration

Here both MRI and MEG data are obtained from the same subject, using fiducial markers.

Biologically interpretable results of localization, obtained under this condition, point at the possible applicability of the proposed method in the studies of the brain ongoing activity.

## Methods

### Computer simulation

The MEG-data were simulated using 61 current dipoles, randomly distributed in space 8 × 8 × 8 cm^3^. The forward problem for the dipole in spherical conductor was solved, generating sinusoidal signal. The time of simulation was equal to 1 min with sampling frequency 1200 Hz. Frequencies changed from 9.5 to 10.5 Hz with the step 0.0167 Hz. Amplitudes were randomly distributed from 10 to 100 fT, corresponding to experimental results for humans in this frequency band. Parameters of the gradiometer for simulation were taken from experimental “noise” collection data. This was obtained by making a 1-min recording under the same conditions as during a MEG recording from a subject, in the absence of the subject (sampling frequency 1200 Hz) in order to estimate the level of noise. The sum of 61 model MEGs was calculated, and the estimated noise MEG was added to account for the noise. Resulting magnetoencephalogram and its multichannel spectrum qualitatively correspond to experimental data for humans in the alpha frequency band.

### Phantom

A current dipole phantom (CTF Systems) was used. This phantom consists of a spherical saline-filled vessel, 13 cm inner diameter, providing an appropriate current flow conductor. Inside this vessel, several current dipoles were installed. Each dipole comprises two gold spheres 2 mm in diameter with a 9 mm center-to-center separation.

### Subjects and data acquisition

MEG recordings were acquired from 10 healthy adults (5 men and 5 women) aged 28–76 years of age (mean 41.8 ± 5.4 years; median age 33.5 years). This study was carried out with the approval of the New York University School of Medicine Institutional Review Board. All subjects gave written informed consent in accordance with the Declaration of Helsinki. Participants were recruited from the New York University Medical Center and the local community. MEG recordings were implemented at the New York University School of Medicine Center for Neuromagnetism (CNM) located at the Bellevue Hospital Center. The subjects were asked to relax but stay awake during each 7-min recording period in 42 10-s trials. Recordings were made during both “eyes closed” (EC) and “eyes open” (EO) conditions. Three fiducial markers were applied (left and right preauricular points, and the nasion) to localize the head during the MEG recording.

MEG recordings were carried out in a mu-metal magnetically shielded room using a 275-channel instrument (CTF Systems) while the subject sat upright (sample rate 600 or 1200 Hz). Recordings were from 275 channels. Artifacts and distant noise were reduced using a 3rd order gradientometer (McCubbin et al., 2004). The activity of the instrument and distant noise were recorded before each session.

### Data analysis

The MEG instrumentation supports simultaneous multichannel recordings of magnetic fields from brain activity generated at discrete time points, thus providing sets of discrete experimental vectors {*b*_*k*_}. Instantaneous field value *b*_*k*_(*i*) is registered at the time moment τ_*i*_, *i* = 1, …, *L*, τ_1_ = 0. The first step in our methodology is the interpolation of the experimental data in every channel (Boyd, 2001):

(1) $$\begin{array}{l} {\tilde{B}}_{k}\left(t\right)=\frac{\left(t-\tau_{i+1}\right)}{\left(\tau_{i}-\tau_{i+1}\right)}b_{k}\left(i\right)+\frac{\left(t-\tau_{i}\right)}{\left(\tau_{i+1}-\tau_{i}\right)}b_{k}\left(i+1\right),t\in \left[\tau_{i},\tau_{i+1}\right],\\ \;i=1,\ldots ,L-1,k=1,\ldots ,K. \end{array}$$

Interpolation provides the continuous function ${\tilde{B}}_{k}\left(t\right),\;t\in \left[0,T\right],\;T=\tau_{L-}\tau_{1}$, where *T* is the time of measurement, *k* is the channel number.

The multichannel Fourier transform calculates a set of spectra for interpolated functions {${\tilde{B}}_{k}\left(t\right)$}:

(2) $$\begin{array}{l} a_{0k}=\frac{2}{T}\int_{0}^{T}{\tilde{B}}_{k}\left(t\right)dt,\;a_{nk}=\frac{2}{T}\int_{0}^{T}{\tilde{B}}_{k}\cos\left(2\pi \nu_{n}t\right)dt,\\ \;b_{nk}=\frac{2}{T}\int_{0}^{T}{\tilde{B}}_{k}\sin\left(2\pi \nu_{n}t\right)dt, \end{array}$$

Where *a*_0*k*_, *a*_*nk*_, *b*_*nk*_ are Fourier coefficients for the frequency ν_*n*_ in the channel number *k*, and $\nu_{n}=\frac{n}{T}$, *n* = 1, …, *N, N* =, where ν_max_ is the highest desirable frequency. The coefficient *a*_0*k*_ will not be considered hereafter, because the constant field component has no meaning in superconducting quantum interference device (SQUID) measurements.

All spectra are calculated for the whole registration time T, which is sufficient to reveal the detailed frequency structure of the system. The step in frequency is equal to $\Delta \nu =\;\nu_{n}-\nu_{n-1}=\frac{1}{T}$, thus frequency resolution is determined by the recording time. Gaussian quadrature formulas are used to calculate integrals on any interval [0, *T*], so the interpolation (1) makes it possible to optimize frequency grid, changing *T* (Llinás and Ustinin, 2014a,b). If the optimization is not necessary, and the time array τ provides quadrature nodes to calculate integrals with sufficient precision, then the data are used without interpolation. In this study integrals were calculated without interpolation.

Given a precise multichannel spectrum, it is possible to perform the inverse Fourier transform:

(3) $$\begin{array}{l} B_{k}\left(t\right)=\;\overset{N}{\underset{n=1}{\sum }}\rho_{nk}\text{ sin}\left(2\pi \nu_{n}t+\varphi_{nk}\right),\;\nu_{n}=\frac{n}{T},\;N=\nu_{\text{max}}T, \end{array}$$

Where $\rho_{nk}=\sqrt{a_{nk}^{2}+b_{nk}^{2}},\;\varphi_{nk}=atan2\left(a_{nk},b_{nk}\right)$, and *a*_*nk*_, *b*_*nk*_ are Fourier coefficients, found in (2).

Precision of the direct and inverse Fourier transforms, used in our approach, can be illustrated by the fact, that initial MEG is restored from (3) with relative error less than 10^−20^.

In order to study the detailed frequency structure of the brain, we restore multichannel signal at every frequency and analyze the functions obtained. Multichannel signal is restored at frequency ν_*n*_ in all channels:

(4) $$\begin{array}{l} B_{nk\left(t\right)}=\;\rho_{nk}\sin\left(2\pi \nu_{n}t+\varphi_{nk}\right), \end{array}$$

where $t\in \left[0,T_{\nu_{n}}\right],\;T_{\nu_{n}}=\frac{1}{\nu_{n}}$ is the period of this frequency.

If φ_*nk*_ = φ_*n*_, then formula (4) describes a coherent multichannel oscillation and can be written as:

(5) $$\begin{array}{l} B_{nk\left(t\right)}=\;\rho_{nk}\sin\left(2\pi \nu_{n}t+\varphi_{n}\right)=\;{\hat{\rho }}_{nk}\rho_{n}\sin\left(2\pi \nu_{n}t+\varphi_{n}\right), \end{array}$$

where $\rho_{n}=\sqrt{\overset{K}{\underset{k=1}{\sum }}\rho_{nk}^{2}}$ is the amplitude, and ${\hat{\rho }}_{nk}=\frac{\rho_{nk}}{\rho_{n}}$ is the normalized pattern of oscillation.

In multichannel measurements space is determined by positions of channels. If time course does not depend on *k*, we have separation of time and space variables.

The normalized pattern makes it possible to determine the spatial structure of the source from the inverse problem solution, and this structure is constant throughout the entire period of the oscillation. The time course of the field is determined by the function ρ_*n*_sin(2πν_*n*_*t* + φ_*n*_), which is common for all channels, i.e., this source is oscillating as a whole at the frequency ν_*n*_.

The theoretical foundations for the reconstruction of static functional entities (neural circuits, or sources) have been developed (Llinás and Ustinin, 2014a,b). This reconstruction is based on detailed frequency analysis and extraction of the frequencies, having high coherence and similar patterns.

The algorithm of mass precise frequency-pattern analysis was formulated as:

1. Precise Fourier Transform of the multichannel signal.
2. Inverse Fourier Transform—restoration of the signal at each frequency.
3. If the coherence at the particular frequency is close to 1, then use the pattern and frequency as elementary coherent oscillation, see Equation (5).
4. If the restored signal consists of several phase-shifted coherent oscillations, then extract those oscillations:
  - Apply second order blind identification (SOBI) algorithm (Belouchrani et al., 1997) to restored time-series in Equation (4);
  - Select nonzero components;
  - Apply direct Fourier transform to each selected component and calculate amplitude, normalized pattern and phase using Equation (5).

After the fourth step of this analysis, the initial multichannel signal is represented as a sum of elementary coherent oscillations:

(6) $$\begin{array}{r} B_{k}\left(t\right)≅\overset{N}{\underset{n=1}{\sum }}\overset{M}{\underset{m=1}{\sum }}D_{mn}{\hat{\rho }}_{mnk}\text{sin}\left(2\pi \nu_{n}t+\varphi_{mn}\right),\\ \nu_{n}=\frac{n}{T},\;N=\nu_{\text{max}}T,\;m=1,\ldots ,M, \end{array}$$

where M is maximal number of coherent oscillations, extracted at the frequency ν_*n*_.

Each elementary oscillation is characterized by frequency ν_*n*_, phase φ_*mn*_, amplitude *D*_*mn*_, normalized pattern ${\hat{\rho }}_{mnk}$ and is produced by the functional entity having a constant spatial structure.

The method of functional tomography reconstructs the structure of the system from the analysis of the set of normalized patterns $\left\{{\hat{\rho }}_{mn}\right\}$.

The functional tomogram displays a 3-dimensional map of the energy produced by all the sources located at a given point. In order to build a functional tomogram, the space under study is divided into *N*_*x*_ × *N*_*y*_ × *N*_*z*_ elementary cubicles with centers in **r**_*ijs*_. The edge of the cubicle is selected in accordance with desirable precision and/or computational facilities; in this study, it was 1.0 mm for simulated data, 1.5 mm for phantom data, and 3.0 mm for human data. To calculate the energy produced by all the sources located in the center of the cubicle, the set of *L* trial dipoles **Q**_*ijsl*_ is build. The magnetic induction, produced by the trial dipole **Q**_*ijsl*_ located in **r**_*ijs*_, is registered by the probe number *k* with position **r**_*k*_ and direction **n**_*k*_. The *k*-th component $\rho_{ijslk}^{tr}$ of the trial pattern *ijsl* is calculated from the model of a current dipole in a spherical conductor (Sarvas, 1987):

(7) $$\begin{array}{l} \rho_{ijslk}^{tr}=\frac{\mu_{0}}{4\pi F^{2}}\left(\left(\left({\text{Q}}_{ijsl}\times {\text{r}}_{ijs}\right)F-\left({\text{Q}}_{ijsl}\times {\text{r}}_{ijs},\;{\text{r}}_{k}\right)\nabla F\right),{\text{n}}_{k}\right), \end{array}$$

where $F=a\left(ar_{k}+r_{k}^{2}-\left(r_{ijs},r_{k}\right)\right),\nabla F=(a^{2}r_{k}^{-1}+a^{-1}\left(a,r_{k}\right)+2a+2r_{k})r_{k}-\left(a+2r_{k}+a^{-1}\left(a,r_{k}\right)\right)r_{ijs}$, **a** = **r**_*k*_ − **r**_*ijs*_, *a* = |**a**|, *r*_*k*_ = |**r**_*k*_|, |**n**_*k*_| = 1, $\mu_{0}=4\pi \cdot 10^{-0}$. Full set of $\rho_{ijslk}^{tr}$ provides lead field matrix for the particular device (Hämäläinen and Ilmoniemi, 1994; Sekihara and Nagarajan, 2008).

The normalized trial pattern is calculated as:

(8) $${\hat{\rho }}_{ijslk}^{tr}=\frac{\rho_{ijslk}^{tr}}{\left|\rho_{ijsl}^{tr}\right|},\text{where}\left|\rho_{ijsl}^{tr}\right|=\sqrt{\sum_{k=1}^{K}{\left(\rho_{ijslk}^{tr}\right)}^{2}}.$$

All trial dipoles, originating from **r**_*ijs*_, lie in the same plane, orthogonal to **r**_*ijs*_, because the vector product **Q**_*ijsl*_ × **r**_*ijs*_ is nonzero only for those dipoles. Trial dipoles cover the circle in *L*_*max*_ directions with 360∕*L*_*max*_ degrees step, in this study *L*_*max*_ = 8.

The set of normalized trial patterns is then calculated, using (8) for each trial dipole:

(9) $$\begin{array}{l} \left\{\rho_{ijsl}^{tr}\right\},\;i=1,\ldots ,N_{x};j=1,\ldots ,N_{y};s=1,\ldots ,N_{z};\\ \;l=1,\ldots ,L_{max}. \end{array}$$

In this study more than 2.5 million trial patterns were used for each person. Those patterns were produced by trial dipoles, uniformly distributed in the localization space.

For each normalized pattern ${\hat{\rho }}_{mn}$, the following function was calculated, giving the difference between this pattern and one of the trial patterns:

(10) $$\begin{array}{l} \chi \left(i,j,s,l\right)=\;\overset{K}{\underset{k=1}{\sum }}{\left({\hat{\rho }}_{ijslk}^{tr}-{\hat{\rho }}_{mnk}\right)}^{2}, \end{array}$$

where ${\hat{\rho }}_{ijslk}^{tr}$ is a *k*-th component of the trial pattern *jsl* and ${\hat{\rho }}_{mnk}$ is a *k*-th component of the normalized pattern *mn*, *k*—number of channel.

The position and direction of the source producing the pattern ${\hat{\rho }}_{mn}$ were determined by numbers (*I, J, S, L*), providing the minimum to the function χ(*i, j, s, l*) over the variables *i* = 1, …, *N*_*x*_; ; *j* = 1, …, *N*_*y*_; ; *s* = 1, …, *N*_*z*_; ; *l* = 1, …, *L*_*max*_. The minimum of this function was found by the exhaustive search, selecting the smallest value from the whole set of 2.5 millions χ for each ${\hat{\rho }}_{mn}$. Such procedure determines **r**_*IJS*_—the inverse problem solution for the pattern ${\hat{\rho }}_{mn}$, without filtering of channels, or weighting functions.

The energy of this source $D_{mn}^{2}$ is added to the energy produced from the cubicle with the center at **r**_*IJS*_.

Performing this procedure for all normalized patterns: *m* = 1, …, *M*; ; *n* = 1, …, *N*, it is possible to distribute in space the energy of all oscillations from formula (6). The result of such distribution is the Functional Tomogram of the brain, reconstructed from MEG.

## Experimental results

### Computer simulation

The simulated MEG was analyzed by the method proposed in Section Data Analysis. The functional tomogram yielded a 3-dimensional map of energy in the frequency band 9.5–10.5 Hz, distributed in a 8 × 8 × 8 cm^3^ cube (in empty space) with a 1.0 mm resolution. For each frequency, the calculated functional tomogram was compared with the coordinates of simulated current dipole. The average distance between the dipole true position and the center of the elementary cubicle to which this dipole was localized, was estimated as 0.7 ± 0.1 mm.

### Phantom

The phantom was placed in the center of the MEG recording helmet. Three localization coils were placed on the spherical vessel, corresponding to usual head placement (front, left and right side, separated by 90° on the sphere's equator) thus providing the necessary fiducial markers. Three dipoles were activated simultaneously with alternate current from separate generators, at 7.00, 7.83, and 11.00 Hz. The magnetic field produced by the phantom dipoles was recorded for 100 s.

**Figure 1:**
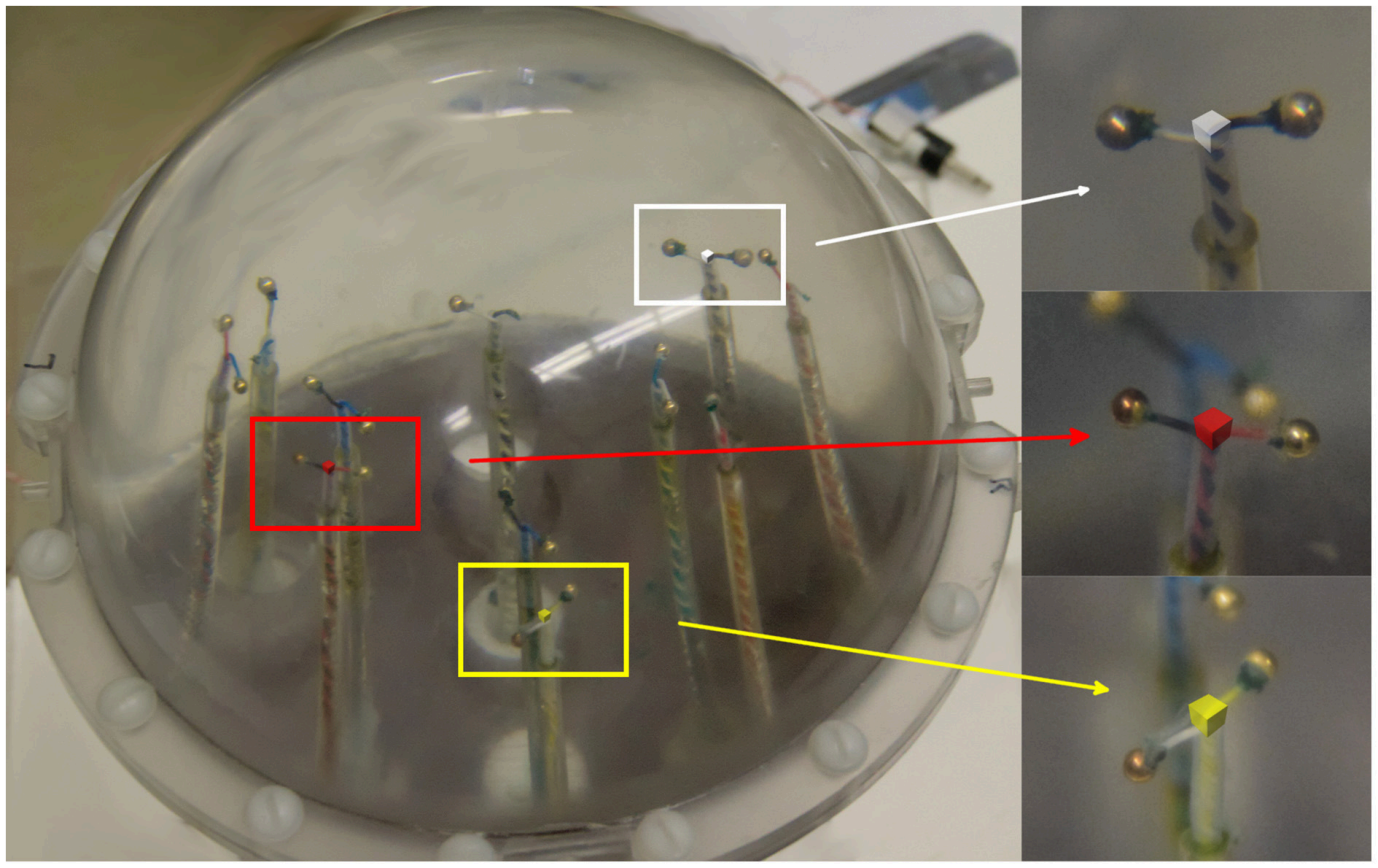
**Functional tomogram superimposed on a photograph of the current dipole phantom (using fiducial markers)**. Three nonzero cubes designate calculated localization of three stimulated dipoles; red (dipole 2: *x* = 25, *y* = −25, *z* = −6), yellow (dipole 6: *x* = −25, *y* = 25, *z* = 38), and white (dipole 11: *x* = −36, *y* = 0, *z* = 14). All coordinates are given in millimeters.

The functional tomogram, calculated as described in Section Methods, yielded a 3-dimensional map of energy in the frequency band 1–40 Hz, distributed in a 10 × 10 × 10 cm^3^ cube (in empty space) with a 1.5 mm resolution. Then the calculated functional tomogram was superimposed on a photograph of the phantom (white, red and yellow cubes 1.5 × 1.5 × 1.5 mm^3^ in Figure 1). All cubes were localized to the centers of the phantom dipoles with an error of less than 1 mm.

### The alpha rhythm

The current method makes it possible to study spontaneous brain resting activity, and to analyze the distribution of sources in the brain. The alpha rhythm was selected for this study since it is the dominant oscillation in healthy adults when the eyes are closed (see Basar, 2012, for a review). In broad terms, the alpha band has been defined as 8–13 Hz brain generated rhythm having, typically, a 9–11 Hz frequency in healthy adults (Nunez et al., 2001). To eliminate differences in the alpha peak across individuals, the individual alpha frequency (IAF) (Klimesch et al., 1999) was determined for each subject.

Let us consider the processing of experimental data set for one subject (#4 in **Figure 3**). Two multichannel spectra were calculated in the frequency band 8–13 Hz, each spectrum contains 2100 frequency peaks in 275 channels:

(11) $$\begin{array}{l} B_{k}\left(t\right)=\sum_{n=n_{min}}^{n_{max}}\rho_{nk}\sin\left(2\pi \nu_{n}t+\varphi_{nk}\right),\;\nu_{n}=\frac{n}{T},\;n_{min}=3361,\\ n_{max}=5460,\;k=1,\ldots ,275 \end{array}$$

**Figure 2:**
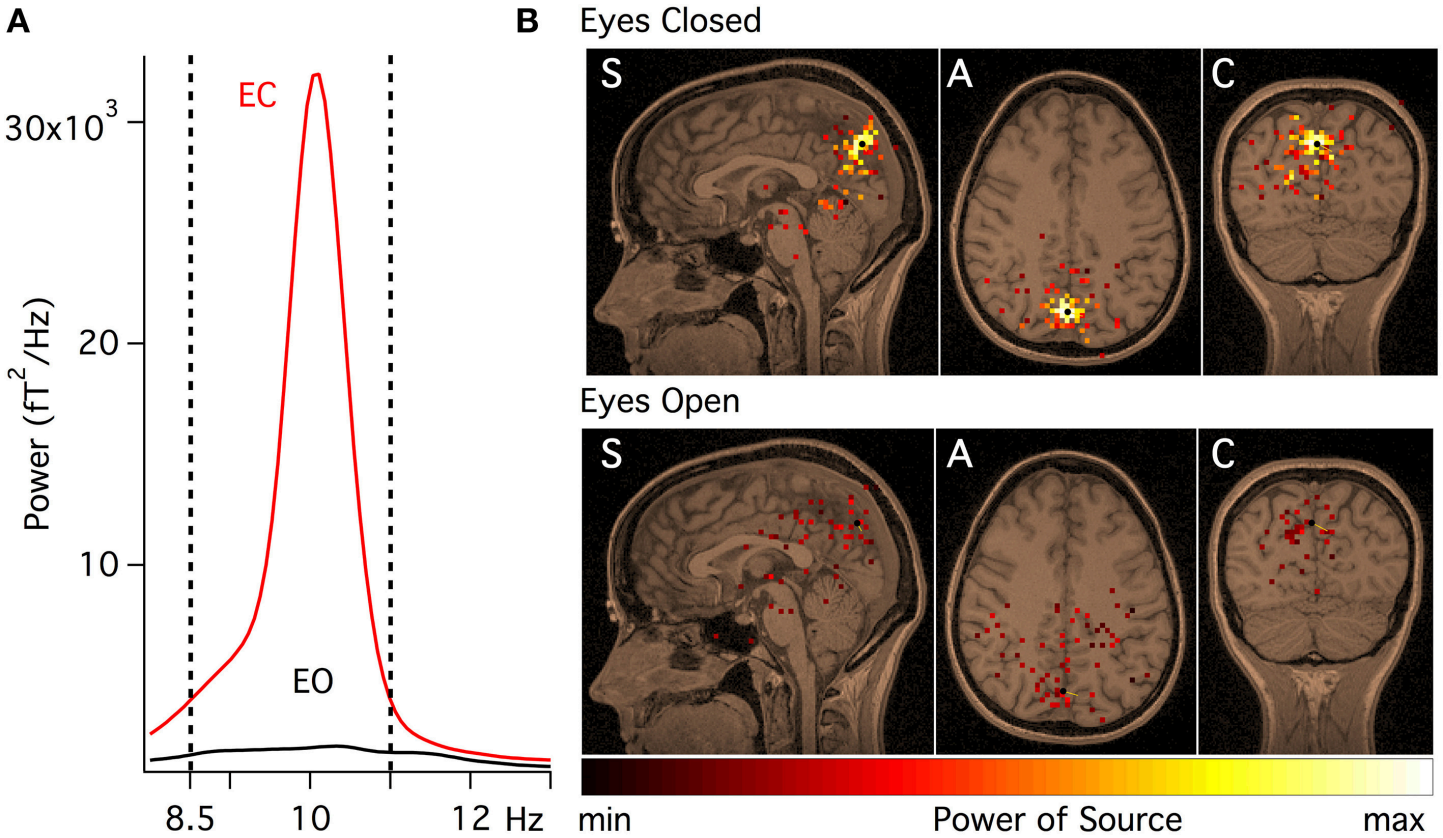
**Alpha peak in a healthy adult subject. (A)** Power spectra of MEG recorded with the eyes closed (EC, red) and with the eyes open (EO, black) while the subject was awake and relaxed. **(B)** Functional tomograms at frequency band 8.5–11 Hz, for MEG recording made with the eyes closed and the eyes open co-registered over the subject's MRI. Standard tomographic sections are shown: sagittal (S), axial (A), and coronal (C). All sections cross at the point denoted by the black marker.

Figure 2A shows the power spectra (calculated using the Welch method) (Welch, 1967) for subject #4 recorded with the eyes open (EO) and the eyes closed (EC). It can be concluded, that those two states demonstrate different spectral features, namely, the spectrum for the EC condition contains a peak near 10 Hz that decreased when the recording was made with subject's eyes open.

From the analysis of Figure 2A, frequency band 8.5–11 Hz was selected for further analysis, as characteristic for EC condition. Source localizations for this band, recorded with the EC and the EO, are shown in Figure 2B co-registered on the subject's MRI. The total energy in the alpha frequency band recorded with the EC was much stronger and was concentrated in a smaller volume, than the corresponding spectral energy generated when the eyes were open during the recording, as would be expected in healthy adults (Nunez et al., 2001).

**Figure 3:**
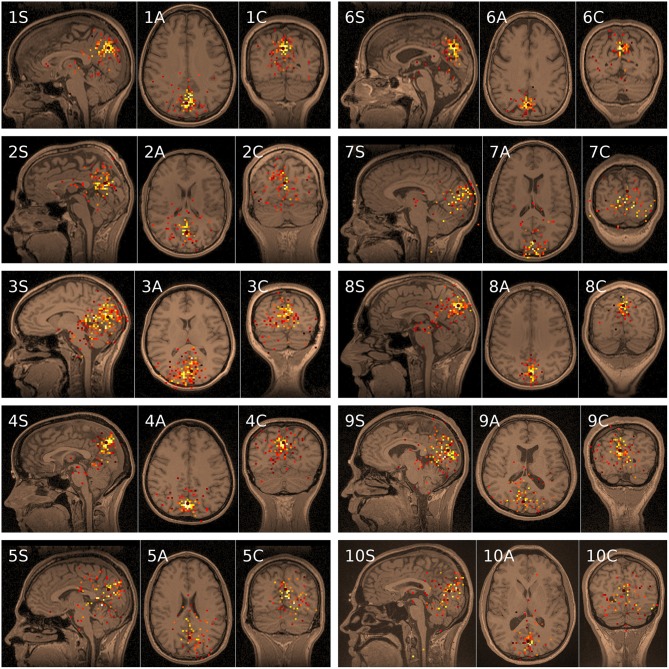
**Functional tomograms of alpha band spontaneous activity co-registered with MRIs for 10 subjects recorded with the eyes closed**. Each tomogram shows three standard tomographic sections S (sagittal), axial (A), and C (coronal).

**Figure 4:**
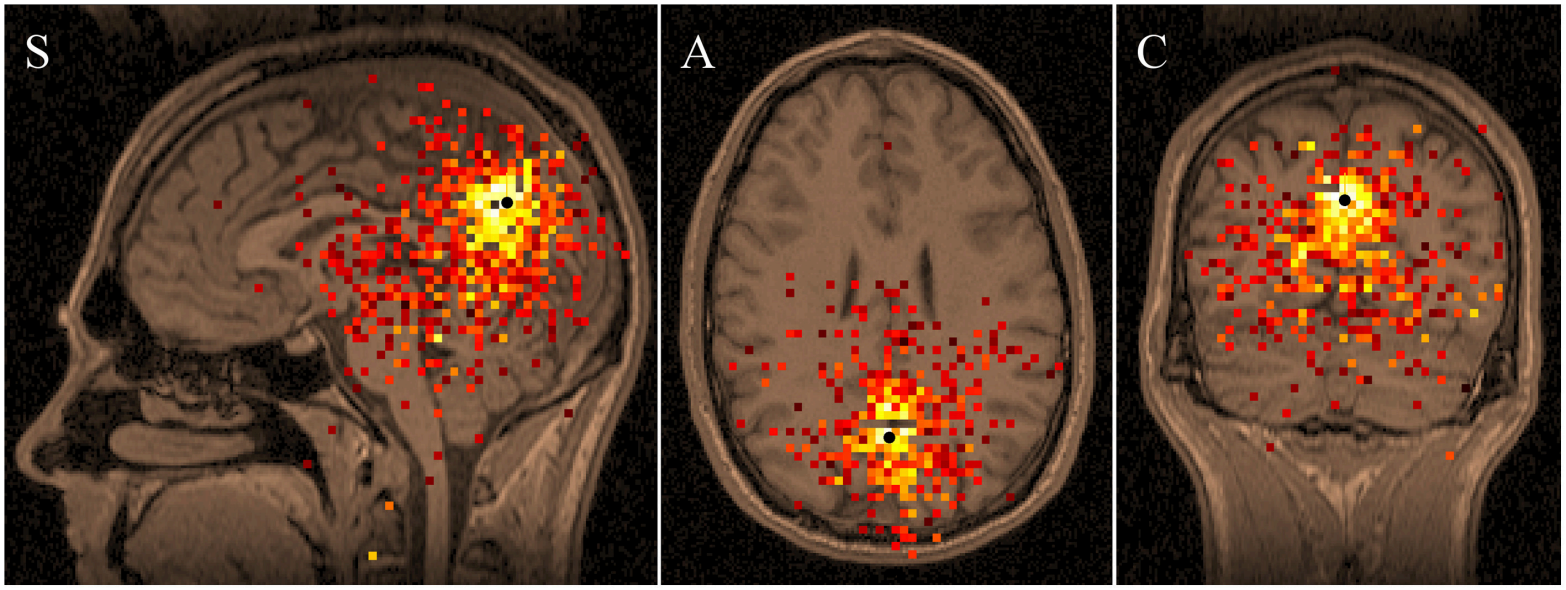
**Average “Eyes Closed” functional tomogram of alpha band activity recorded from 10 individuals, plotted over the MRI from subject #5**. Sagittal (S), axial (A), and coronal (C) tomographic sections are shown.

The same data processing protocol was applied and similar results were obtained for all 10 subjects. Figure 3 shows 10 functional tomograms with corresponding MRIs for the EC condition. For each subject three tomographic sections (Figure 4, S, sagittal; A, axial; C, coronal) are shown. The sections transect the same point in space (black marker) located in the region of the strongest source. Such sources were denoted by white voxels in the functional tomogram, in accordance with legend for Figure 2B. Presentation of the data was performed in the program environment MEGMRIAn (Ustinin et al., 2014). The spatial resolution for the MRI is equal to 1 mm, for the functional tomogram it is 3 mm. Eight directions were used for trial dipoles in every point of the spatial grid, as explained in Section Subjects and Data Acquisition, Equations (7) and (8).

Figure 4 shows the superposition of the 10 functional tomograms shown on the MRI from subject #5 in Figure 3. This summation was performed in the head coordinate system, common for all functional tomograms. Note, that regardless of individual variances, the alpha rhythm energy distribution displays general tendency to be located in occipital and posterior parietal lobes.

### Resolution of the method

There are two kinds of resolution in this approach: frequency resolution and space resolution. Frequency resolution $\Delta \nu =\nu_{n}-\nu_{n-1}=\frac{1}{T}$ is determined by the time of measurement, on condition that Fourier integrals for the full time of measurement (2) are calculated precisely. It is reflecting the fundamental fact: the longer one registers time series, the better one determines frequency structure of the system. In this study of spontaneous activity *T* was equal to 420 s, thus providing 420 frequencies per one Hz.

Spatial resolution has no theoretical limitations in this approach. Note, that the functional tomograms were calculated with spatial resolution of 1.0 mm for simulated data, 1.5 mm for phantom data, and 3.0 mm for human data. These differences were determined by computational limitations and followed from the usage of a space of 8 × 8 × 8 cm^3^ for simulated data, of 10 × 10 × 10 cm^3^ for phantom and of 25 × 25 × 25 cm^3^ for human functional tomograms. By increasing computer memory, one can obtain a higher spatial resolution. Precision of localization can be estimated from the known dipoles positions in cases of simulated and physical dipoles. It was found, that precision ≈ 0.7 of resolution.

For each elementary coherent oscillation, found in (6), unique dipolar source is localized by selection of the best trial source from 2.5 million, distributed in the whole space of MRI. It means that no a-priori limitations are used for the location of sources, and their combined representation with MRI may provide new information. Using normalized patterns, one can obtain localization of weak sources, if they are extracted from Fourier analysis, with precision equal to the precision of localization of strong sources. It opens new possibilities to study deep brain sources.

Two and more oscillations can have common position and direction, thus providing the spectrum of the particular source (partial spectrum) (see Llinás and Ustinin, 2014a,b). The inverse Fourier transform gives time series, produced by this source. Selecting two or more such sources, one can study their connectivity, using methods described in Greenblatt et al. (2012).

## Discussion

A novel method to implement the analysis of human brain activity addressed as functional tomography is introduced. This novel methodology was used to calculate the spatial distribution of brain activity power sources recorded with an MEG instrument. This method is free of arbitrary parameters, it is computationally stable, and it is free from matrix inversion requirements. Computational demands are reasonable for modern computers. Thus, a functional tomogram may be implemented in 20 min on a computer with 2.4 GHz 4-cores Haswell CPU and 16 GB RAM.

Functional tomograms were obtained for alpha rhythm from multichannel MEG data. These functional tomograms demonstrate individual variances of the power spatial distribution, generally corresponding to our present knowledge concerning the alpha rhythm localization in the occipital and posterior parietal lobes (Nunez et al., 2001; Basar, 2012). It can be concluded, therefore, that the functional tomography method, based on magnetic-encephalograms analysis, can determine spontaneous brain activity sources.

A fundamental advantage of this framework lies in the fact, that all recorded data is fully utilized.

Method of functional tomography can be applied to the diagnostics of activity in the whole brain and in broad frequency band, revealing areas of abnormally high or abnormally low activity.

### Conflict of interest statement

The authors declare that the research was conducted in the absence of any commercial or financial relationships that could be construed as a potential conflict of interest.
